# Comparative analysis of cytosolic and mitochondrial ATP synthesis in embryonic and postnatal hippocampal neuronal cultures

**DOI:** 10.3389/fnmol.2012.00102

**Published:** 2013-01-10

**Authors:** Alexander M. Surin, Serguei Khiroug, Lubov R. Gorbacheva, Boris I. Khodorov, Vsevolod G. Pinelis, Leonard Khiroug

**Affiliations:** ^1^Neuroscience Center, University of HelsinkiHelsinki, Finland; ^2^Institute of General Pathology and Pathophysiology, Russian Academy of Medical SciencesMoscow, Russia; ^3^Scientific Center for Children's Health, Russian Academy of Medical SciencesMoscow, Russia; ^4^Faculty of Biology, Lomonosov Moscow State UniversityMoscow, Russia

**Keywords:** OxPhos, F1Fo-ATPase, TMRM, adenosine triphosphate, fluorescent protein

## Abstract

ATP in neurons is commonly believed to be synthesized mostly by mitochondria via oxidative phosphorylation. Neuronal mitochondria have been studied primarily in culture, i.e., in neurons isolated either from embryos or from neonatal pups. Although it is generally assumed that both embryonic and postnatal cultured neurons derive their ATP from mitochondrial oxidative phosphorylation, this has never been tested experimentally. We expressed the FRET-based ATP sensor AT1.03 in cultured hippocampal neurons isolated either from E17 to E18 rat embryos or from P1 to P2 rat pups and monitored [ATP]c simultaneously with mitochondrial membrane potential (ΔΨm; TMRM) and NAD(P)H autofluorescence. In embryonic neurons, transient glucose deprivation induced a near-complete decrease in [ATP]c, which was partially reversible and was accelerated by inhibition of glycolysis with 2-deoxyglucose. In the absence of glucose, pyruvate did not cause any significant increase in [ATP]c in 84% of embryonic neurons, and inhibition of mitochondrial ATP synthase with oligomycin failed to decrease [ATP]c. Moreover, ΔΨm was significantly reduced by oligomycin, indicating that mitochondria acted as consumers rather than producers of ATP in embryonic neurons. In sharp contrast, in postnatal neurons pyruvate added during glucose deprivation significantly increased [ATP]c (by 54 ± 8%), whereas oligomycin induced a sharp decline in [ATP]c and increased ΔΨm. These signs of oxidative phosphorylation were observed in all tested P1–P2 neurons. Measurement of ΔΨm with the potential-sensitive probe JC-1 revealed that neuronal mitochondrial membrane potential was significantly reduced in embryonic cultures compared to the postnatal ones, possibly due to increased proton permeability of inner mitochondrial membrane. We conclude that, in embryonic, but not postnatal neuronal cultures, ATP synthesis is predominantly glycolytic and the oxidative phosphorylation-mediated synthesis of ATP by mitochondrial F1Fo-ATPase is insignificant.

## Introduction

A variety of pathological conditions, such as ischemia, stroke, or traumatic injury, deprives neurons of oxygen and glucose supply, thus leading to depletion of intracellular adenosine triphosphate (ATP) levels (see for reviews Ames, 2000; Duchen, 2004; Iijima, 2006; Duchen and Szabadkai, 2010; Gleichmann and Mattson, 2011; Gouriou et al., 2011). Intracellular ATP is a ubiquitous “energy currency” that is crucial for energy-dependent functions in neurons including homeostasis of transmembrane ion gradients (Ames, 2000; Beal, 2000; Nicholls and Budd, 2000). Suppression of ATP synthesis via glycolysis and oxidative phosphorylation (OxPhos) causes deregulation of transmembrane gradients for key ions including Na^+^, K^+^, and Ca^2+^ that are maintained by plasmalemmal and intracellular ATPases, the major consumers of ATP in neurons.

Primary neuronal cultures have been widely used as a convenient (and often indispensable) model for investigation of molecular and cellular mechanisms of brain pathologies, such as ischemia or excitotoxic effects of high-dose glutamate. Hippocampal neurons are usually cultivated upon dissociation from either embryonic (E17–E18) or postnatal (P1–P2) rodent brain. It is commonly assumed that, after 1 or 2 weeks in culture, embryonic, and postnatal neurons have similar properties in terms of their response to oxidative stress, toxic doses of glutamate or glucose deprivation, i.e., to those pathology-modeling conditions that impose a heightened demand on neuronal energy suppliers (see for example, Wang and Thayer, 1996; Sattler et al., 1998; Ruiz et al., 1998; Vergun et al., 1999, 2003). Until now, however, no published work has reported a direct comparison of cytosolic ATP concentration ([ATP]c) levels in postnatal vs. embryonic cultured neurons. Such a comparison is timely and relevant, in view of the widespread use of both types of cultures and considering the growing body of evidence that the cascade of molecular and cellular modifications occurring at birth strongly affects bioenergetics of neurons in neonatal brain (Lust et al., 2003; Illsinger and Das, 2010).

To our knowledge, there are only two studies in which luciferase bioluminescence imaging have been used to evaluate changes in [ATP]c of individual cultured neurons (Ainscow et al., 2002; Mironov, 2007). This method has not been widely used, apparently due to the stringent requirements for equipment to be sensitive enough to detect weak bioluminescence signals. In addition to bioluminescence, fluorescence of a Mg^2+^-sensitive indicator mag-Fura can be used for measuring [ATP]c, albeit only under such conditions that do not cause any measurable changes in [Ca^2+^]_*i*_ (Abramov and Duchen, 2010).

The recently introduced genetically encoded ATP sensors based on fluorescent protein pairs have made it possible, for the first time, to monitor changes in either [ATP]c or in the ATP/ADP ratio in single cells and organelles (Berg et al., 2009; Imamura et al., 2009; Nakano et al., 2011) employing conventional epifluorescence microscopy. Here, we used the AT1.03 sensor constructed from two fluorescent proteins (blue and yellow-green) that are linked with a ATP-binding polypeptide (Imamura et al., 2009). The AT1.03 sensor translates changes in [ATP]c into a conformational change that modulates the efficiency of fluorescence resonance energy transfer (FRET) between the two proteins, thus changing measurably the spectral properties of the sensor. Expression of AT1.03 in cytosol of cultured hippocampal neurons allowed us, for the first time, to perform time-lapse measurement of [ATP]c dynamics in individual neurons in response to glucose deprivation, glycolysis blockade and/or manipulations with mitochondrial ATP synthesis. We conclude that, in 84% of hippocampal neurons cultivated from E17 to E18 rat embryos, the mitochondrial ATP synthase (F1Fo-ATPase) does not produce ATP, but instead consumes it in order to maintain mitochondrial membrane potential (ΔΨm). In contrast, mitochondria of the neurons cultivated from neonatal rat pups showed evidence of ATP synthesis by F1Fo-ATPase as expected.

## Results

**Figure 1:**
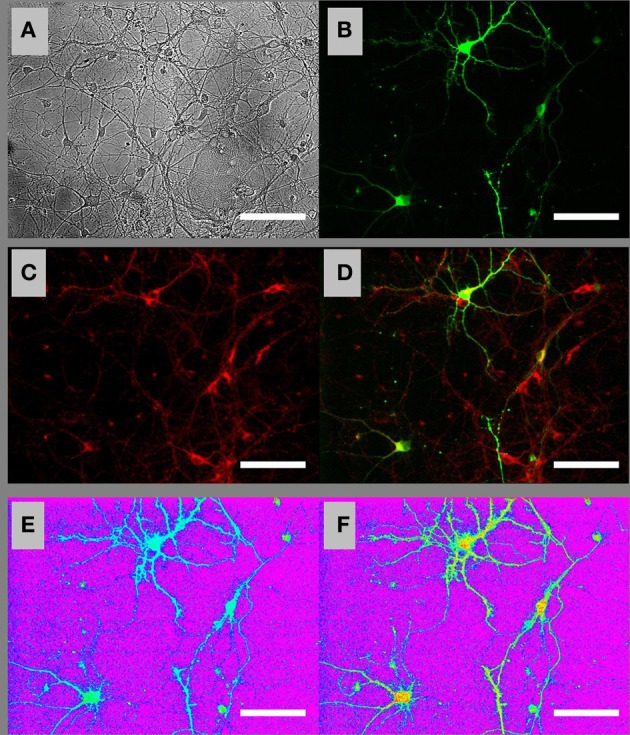
**Ratiometric fluorescence imaging of embryonic cultured hippocampal neurons expressing AT1.03 and loaded with a mitochondrial membrane potential sensitive dye TMRM. (A)** Bright-field image of cells in the visual field. **(B)** Fluorescence image of the same field showing AT1.03 at the acceptor wavelength (excitation 500 nm, emission 535 nm). **(C)** Fluorescence image of TMRM loaded both in the AT1.03-expressing neurons and in the surrounding non-transfected cells. **(D)** Overlay of AT1.03 from **(B)** (green) and TMRM from **(C)** (red). **(E)** Ratiometric AT1.03 image under resting conditions. The image was digitally constructed by diving an F436 image (excitation 436 nm, emission 535 nm) by the nearly-simultaneously acquired F500 image (excitation 500 nm, emission 535 nm). **(F)** Ratiometric AT1.03 image under Gluc deprivation conditions. The pseudo-color palette in **(E)** and **(F)** has been selected so that purple corresponds to the cell-bearing substrate, blue–green colors correspond to the baseline (high) levels of [ATP]c, and “warmer” colors (yellow–red) correspond to progressive depletion of [ATP]c. Scale bars: 50 μm.

To perform time-lapse fluorescence microscopic imaging of [ATP]c in individual neurons, we transfected embryonic and postnatal hippocampal cultures with the ATP sensor AT1.03 (Imamura et al., 2009) and loaded them with a widely used ΔΨm-sensitive dye TMRM. Figure 1 shows bright field **(A)** and fluorescence **(B–D)** images of a visual field in a culture of embryonic hippocampal neurons loaded with TMRM (red in Figures 1C,D). A fraction of cells that were morphologically identified as neurons expressed AT1.03 sensor (green in Figures 1B,D). Figures 1E,F show [ATP]c distribution in AT1.03-expressing embryonic neurons, with [ATP]c encoded on a color scale from blue-green (high [ATP]c, low F436/F500 ratio) to yellow-read (low [ATP]c, high F436/F500 ratio), on the purple background corresponding to the cell-bearing substrate and cells lacking AT1.03 expression.

**Figure 2:**
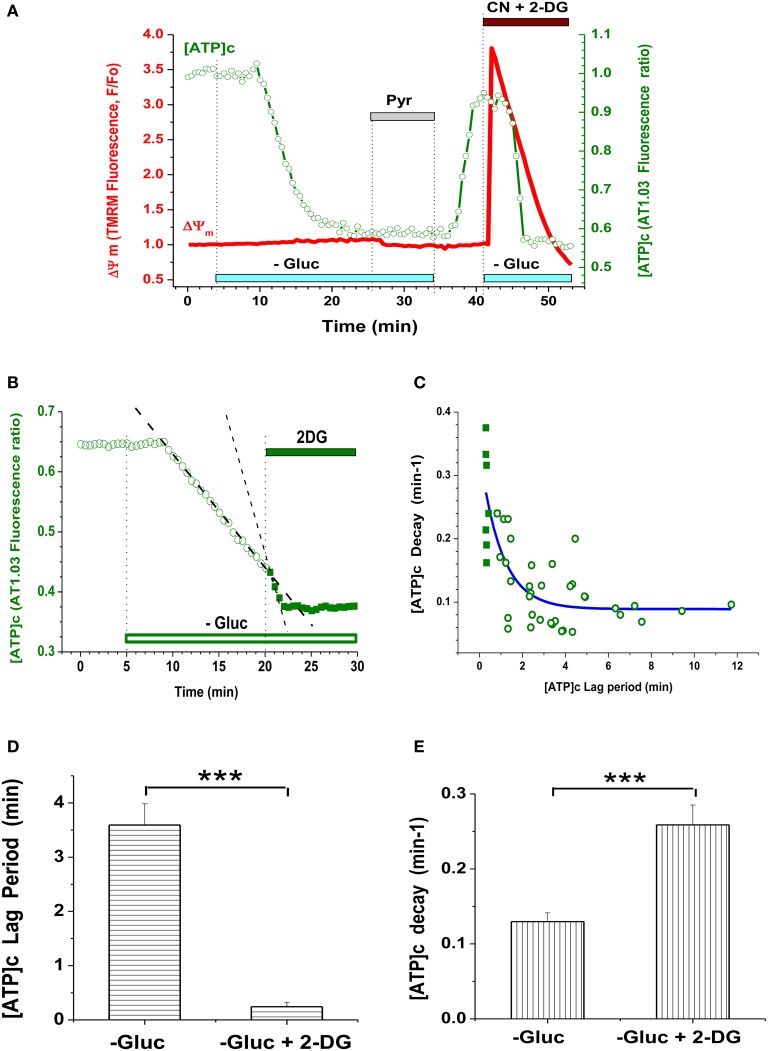
**Glycolysis inhibition in embryonic neurons caused depletion of [ATP]c but had little effect on ΔΨm. (A)** Simultaneous measurement of AT1.03 ratio (F436/F500; [ATP]c, green trace, right axis) and TMRM signal (ΔΨm, red trace, left axis) during inhibition of glycolysis and/or mitochondrial respiration. Gluc deprivation is indicated with light-blue bars, Pyr (10 mM) application with a gray bar, 3 mM NaCN and 5 mM 2-DG (CN + 2-DG) application with a brown bar. **(B)** Example of the accelerating effect of glycolysis blockade with 2-DG on the time course of [ATP]c decrease during Gluc deprivation. Application of 2-DG is indicated with a green bar. Linear fit of the [ATP]c trace in prior to and during 2-DG application is shown as dashed lines. **(C)** Correlative plot of the [ATP]c decay rate (min^−1^) vs. the lag period of [ATP]c decrease induced by Gluc deprivation, as shown in **(A)**. Open symbols: Gluc deprivation alone; filled symbols: Gluc deprivation along with 2-DG. Blue fitting curve was iteratively calculated using the following equation: *Y* = A*exp(-*X*) + *Yo*, where *Y* is the [ATP]c decay rate, *A* is a constant value, *Yo* is an asymptotic rate level, and *X* is the lag period. **(D)** Average lag period of [ATP]c decrease induced by Gluc deprivation alone (left bar) or in combination with 2-DG (right bar). The significant shortening of the lag period by 2-DG is indicated with ^***^ (*P* < 0.001; *n* = 38 and 10). **(E)** Average [ATP]c decay rate during Gluc deprivation alone (left bar) or in combination with 2-DG (right bar). The significant acceleration of the [ATP]c decay by 2-DG is indicated with ^***^ (*P* < 0.001; *n* = 24 and 8).

Removal of glucose (Gluc) from the buffer perfusing embryonic cultured neurons resulted in a marked reduction in [ATP]c (Figure 1F) compared to its pre-deprivation level. The time course of [ATP]c changes had a characteristic pattern exemplified in Figure 2A (green trace): the F436/F500 ratio remained at baseline level for a certain lag period, which ranged between 1 and 10 min, and then dropped abruptly. In Gluc-free buffer, the AT1.03 ratio decreased on average by a factor of 1.62 ± 0.12, i.e., from the normalized base level of 1.0 to 0.62 ± 0.03, or by 38.0 ± 3.0% (*P* < 0.001; *n* = 39 neurons from culture dishes plated on 12 separate cultivation days). Simultaneous monitoring of TMRM signal in the same cells revealed that application of Gluc-free buffer caused only a small decrease in the TMRM signal (by 3.8 ± 0.8%; *n* = 39; *P* < 0.001; see red trace in Figure 2A). The [ATP]c remained at a low steady-state level for as long as extracellular Gluc was absent.

To test whether Gluc deprivation *per se* had decreased [ATP]c to a lowest detectable level in embryonic hippocampal cultured neurons, we applied a combination of 2-DG and cyanide in the Gluc-free medium (Figure 2A, green trace), to ensure that: (1) glycolytic synthesis of ATP is fully blocked and (2) mitochondrial ATPase is only able to hydrolyze ATP, but not to synthesize it (Nicholls and Budd, 2000). Blockade of both glycolitic and oxidative ATP synthesis caused only a marginal decrease (by 2.5 ± 1.0%, *n* = 33) in the AT1.03 ratio from the steady-state level already achieved by Gluc deprivation alone. Thus, Gluc deprivation of embryonic neurons caused a near complete depletion of [ATP]c (i.e., a decrease by 97.5 ± 1.0% of the total dynamic range of the AT1.03 ratio). This finding suggests that, in the absence of extracellular Gluc, embryonic hippocampal neurons quickly lost their ability to produce ATP, at least in such quantities that would be detectable by means of AT1.03 (i.e., above 0.3 mM; Imamura et al., 2009).

Restitution of Gluc in the perfusion buffer readily returned [ATP]c to a level nearly matching the pre-deprivation baseline (Figure 2A, green trace). The increase in the AT1.03 ratio constituted 34.2 ± 1.9% (*P* < 0.01; *n* = 9), reaching ~90% of the value by which AT1.03 ratio decreases in glucose-free buffer (on average, 38.0 ± 1.0%). Similarly to Gluc removal, re-addition of Gluc had little effect on ΔΨm (Figure 2A, red trace). This lack of effect cannot be attributed to insufficient sensitivity of the TMRM imaging assay, because blockade of mitochondrial respiratory chain by sodium cyanide (NaCN, 3 mM) performed at the end of the experiment reliably triggered an immediate strong depolarization of ΔΨm (red trace in Figure 2A). On average, NaCN increased the TMRM signal by 338 ± 24% (*n* = 38; *P* < 0.001). These findings suggest that, during Gluc deprivation, mitochondria had a sufficient amount of pyruvate (or an alternative substrate) for tricarboxylic acid cycle (TCA) to maintain the ΔΨm at a relatively stable level.

The duration of the lag period preceding the abrupt drop in [ATP]c varied widely. This likely reflects variability in the rate of Gluc metabolism between individual cells. Another varying factor may be the abundance of intracellular storage of Gluc, as well as of ATP and glycogene. Indeed, we found that blockade of Gluc metabolism with 2-desoxiglucose (2-DG; 5 mM) on top of Gluc deprivation dramatically shortened the lag period and strongly accelerated the rate of [ATP]c decrease (Figures 2B–D).

**Figure 3:**
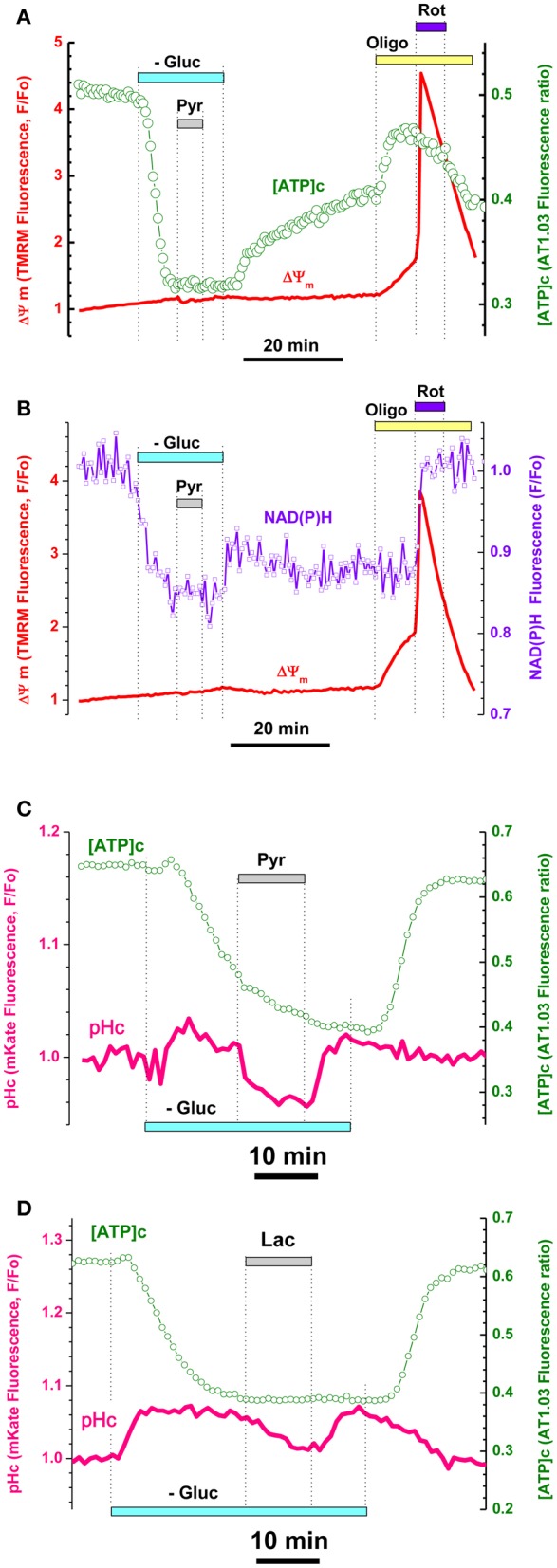
**Pyruvate and lactate failed to increase [ATP]c, ΔΨm, and NAD(P)H despite their penetration into cytosol of hippocampal embryonic neurons. (A)** Simultaneous measurement of [ATP]c (green trace, right axis) and ΔΨm (red trace, left axis) in neuron expressing AT1.03 and loaded with TMRM. Gluc-free buffer application is indicated with a light-blue bar, pyruvate (Pyr, 10 mM) with a gray bar, Oligo (5 μg/mL) with a yellow bar, rotenone (Rot, 2 μM) with a dark blue bar. **(B)** Simultaneous measurement of averaged NAD(P)H autofluorescence (blue trace, right axis) and ΔΨm (red trace, left axis) in seven non-transfected neurons located in the same field of view as those shown in **(A)**. **(C,D)** Intracellular acidification accompanying Pyr **(C)** an lactate (Lac) **(D)** application (both 10 mM) in an embryonic neurons co-expressing AT1.03 (green traces, right axis) and cytosolic pH-sensitive red fluorescence protein mKate (pink traces, left axis). Pyr and Lac application induced a downward shift in mKate fluorescence, consistent with the efficient Pyr/Lac transport causing acidification of cytosol. Pharmacological manipulations—same as in **(A)**.

**Figure 4:**
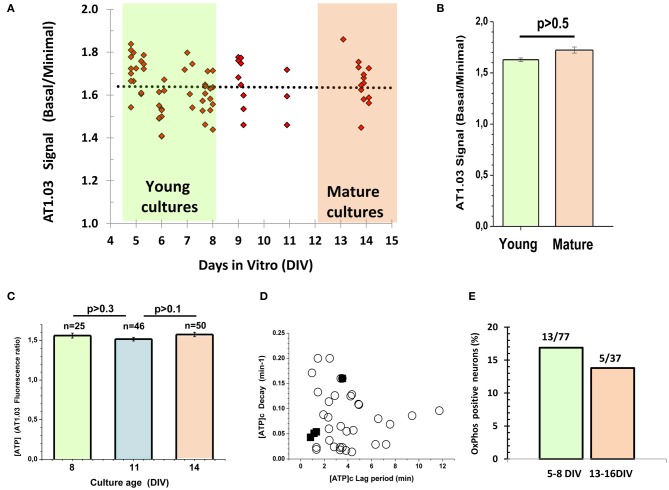
**Lack of correlation between neuronal metabolic parameters and maturity of embryonic cultured neurons. (A)** Plot of time-dependence of the ratio between (1) the AT1.03 signal at the very beginning of the experiment and (2) the minimal signal observed upon suppression of both glycolytic and mitochondrial ATP synthesis. **(B)** The average values for young (5–8 DIV) and mature (13–14 DIV) cultures. **(C)** The averaged ratiometric signals of AT1.03 obtained at 8, 11, and 14 DIV. **(D)** The lag period and the rate of [ATP]c fall after removal of Gluc from the buffer in the hippocampal neurons cultured from embryos. Note that kinetic parameters of [ATP]c changes in the mature cultures (filled squares) are well in the range of typical changes observed in young cultures (open circles) (data from Figure 2C). **(E)** The proportion of neurons in which pyruvate increased [ATP]c in the Gluc-free buffer.

**Figure 5:**
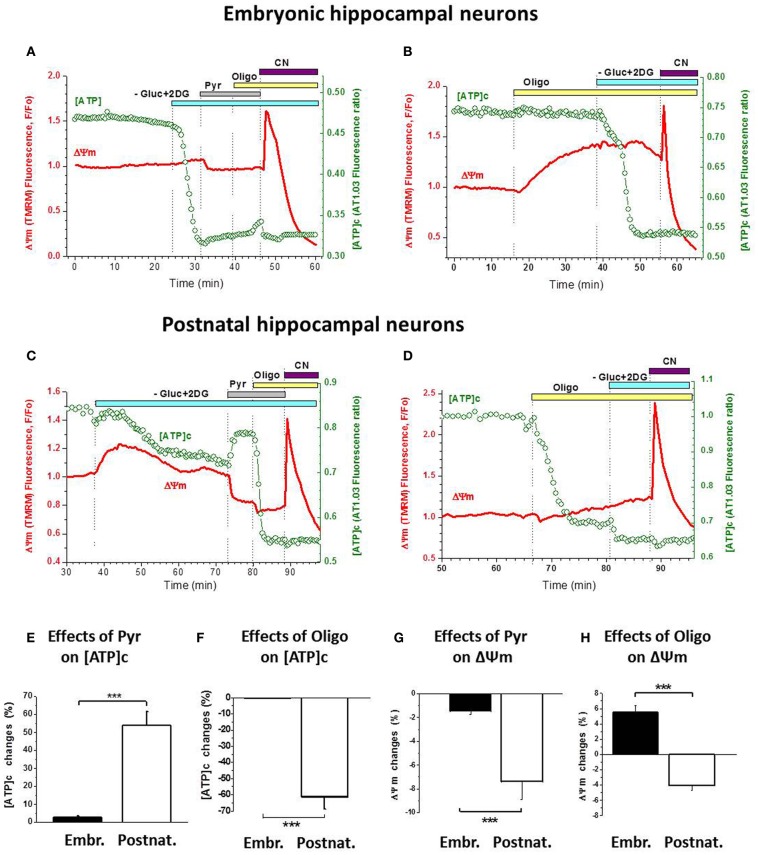
**Postnatal hippocampal cultured neurons, unlike the embryonic ones, have a functional ATP synthesis by F1Fo-ATPase. (A)** Changes in [ATP]c (green trace, right axis) and ΔΨm (red trace, left axis) induced in embryonic neurons by sequential application of Gluc-free buffer containing 2-DG (light-blue bar), 10 mM Pyr (gray bar), 5 μg/mL Oligo (yellow bar), and 3 mM NaCN (purple bar). **(B)** Changes in the above parameters induced in embryonic neurons by application of 5 μg/mL Oligo (yellow bar) followed by Gluc-free buffer containing 2-DG (light-blue bar), and 3 mM NaCN application (purple bar). **(C)** Changes in the above parameters induced in postnatal neurons by the same manipulations as in **(A)**. **(D)** Changes in the above parameters induced in postnatal neurons by the same manipulations as in **(B)**. **(E,F)** Statistical analysis of the changes in [ATP]c induced in embryonic (left bars) and postnatal (right bars) neurons by 10 mM Pyr **(E)** or 5 μg/mL Oligo **(F)**. **(G,H)** Statistical analysis of the changes in ΔΨm induced in embryonic (left bars) and postnatal (right bars) neurons by 10 mM Pyr **(G)** or 5 μg/mL Oligo **(H)**. Statistical significance of the difference between embryonic and postnatal neurons is indicated in **(E–H)** with ^***^ (*P* < 0.001).

We hypothesized that application of pyruvate (Pyr) during Gluc deprivation would restore [ATP]c and increase ΔΨm, because pyruvate (normally provided by glycolysis) is a major substrate for TCA and its addition at 10 mM should boost mitochondrial ATP synthesis (Vergun et al., 2003; Khodorov et al., 2012). To our surprise, a 5–15 min application of 10 mM Pyr (gray bar on Figures 2A, 3A,C, 5A, Appendix Figure A3) to Gluc-deprived embryonic neurons had little if any effect on [ATP]c in ~84% neurons (96/114 neurons, 43 experiments; see Figure 4E). The Pyr effect on [ATP]c in these 96 cells was insignificant and constituted on average a 2.8 ± 0.7% increase in the AT1.03 ratio (*P* > 0.5; *n* = 20). Pyr also induced a modest, but significant hyperpolarization of mitochondria, as evident from the TMRM signal decrease (Figure 2A, red trace, see also Figures 3A,C and 5A) on average by 1.5 ± 0.3% (*P* < 0.001; *n* = 38). Consistently with the Pyr results, application of lactate (Lac) at 10 mM had no effect on [ATP]c (Figure 3D). These results strongly suggest that OxPhos was not active in the embryonic hippocampal neurons.

**Figure A1:**
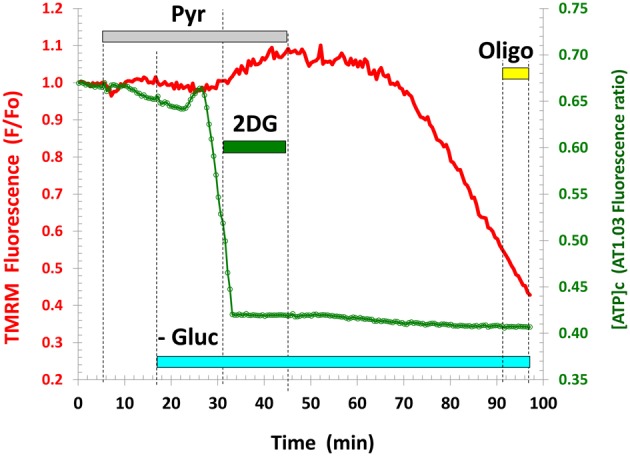
**Application of pyruvate (Pyr) to the resting neurons neither increased the basal [ATP]c, nor prevented its drop following glucose removal.** All agent concentrations are the same as in Figure 1. Similar results were observed in 10 neurons (3 separate experiments).

The inability of Pyr (or Lac) to increase [ATP]c during Gluc deprivation was not related to any irreversible modifications of cellular functions, because restitution of Gluc in the buffer resulted in a near-complete recovery of [ATP]c (Figure 2A, see also Figures 3C,D, Appendix Figure A3). To further verify that Gluc deprivation did not perturb cellular functions, we exposed resting neurons to Pyr prior to and during Gluc withdrawal in a separate set of experiments. The Pyr application neither increased the basal [ATP]c nor occluded the drop in [ATP]c induced by the following Gluc removal (Figure A1). The lack of Pyr effect on the basal [ATP]c at rest indicates that cells do not increase [ATP]c above the level they need. Therefore, in all subsequent experiments Pyr (or Lac) was added only after the [ATP]c decrease caused by removal of Gluc or blockade of glycolysis by 2-DG.

It is known that TCA supplies substrates for the respiratory chain in form of NADH for complex I and succinate for complex II. Mitochondrial NAD(P)H level changes can be estimated from intracellular autofluorescence and has long been used as an important and convenient readout of cellular metabolic status (Chance and Hollunger, 1960; Duchen et al., 2003; Pellerin and Magistretti, 2004; Ying, 2006). We therefore monitored endogenous NAD(P)H fluorescence as an averaged signal from non-transfected cells located in the same visual field as the cells expressing ATP1.03, and compared the time course of simultaneously recorded changes in NAD(P)H and ΔΨm induced by Gluc deprivation and pharmacological manipulations (Figure 3B; see also Appendix Figure A3B).

Gluc removal induced a rapid decrease in NAD(P)H (Figure 3B), which developed in parallel to the decrease in [ATP]c (Figure 3A, see also Appendix Figure A3). At the time when both NAD(P)H fluorescence and AT1.03 ratio were at their lowest plateau levels, we added Pyr (10 mM) and observed no changes in either parameter (respectively green and violet traces in Figures 3A,B). The TMRM signals revealed moderate in response to Pyr application (red traces in Figures 3A,B; statistics in Figure 5G). Washout of Pyr and Lac and restitution of extracellular Gluc resulted in recovery of [ATP]c nearly to the pre-deprivation level.

A possible alternative interpretation of this finding is that exogenous Pyr (and/or Lac) failed to enter the cells through plasma membrane. To evaluate the efficiency of Pyr transmembrane transport in embryonic neurons, we cotransfected the cells with AT1.03 and a red fluorescent protein mKate, which is sensitive to pH changes in a physiological range (pK = 6.0; Shcherbo et al., 2007). Indeed, Pyr and Lac are known to be transported into cells by monocarboxylic acid transporters (Halestrap and Price, 1999; Pierre and Pellerin, 2005) along their transmembrane concentration gradients, and the associated acid influx brings down the intracellular pH. As shown in Figures 3C,D, both Pyr and Lac application induced an immediate downward shift in mKate fluorescence (pink traces) consistent with a decrease in pH. The pH decrease persisted until Pyr or Lac washout, after which pH was quickly restored to its pre-Pyr or pre-Lac level. In contrast to pH, the [ATP]c did not show any changes in response to addition or removal of Pyr (green traces in Figures 3C,D). On average, in three experiments the decrease in mKate signal constituted 6.0 ± 0.8% and was statistically significant (*P* < 0.05; *n* = 12). These data suggest that lack of Pyr and Lac effect on [ATP]c in embryonic hippocampal neurons is not due to restricted transmembrane transport of Pyr or Lac, but rather due to inability of these cells to make use of these substrates for OxPhos-mediated production of ATP.

Blockade of mitochondrial ATPase with oligomycin (Oligo; 5 μg/mL) at the time when [ATP]c recovery was still incomplete induced a significant increase in the AT1.03 ratio (Figure 3A, green trace; on average by 29.8 ± 5.3%; *P* < 0.01, *n* = 6). Such a paradoxic Oligo-induced surge in [ATP]c indicates that mitochondrial ATP synthase was working in a reverse mode, i.e., acted as an ATPase consuming residual ATP (Nicholls and Budd, 2000). Earlier it has been shown that application of Oligo to intact cultured nerve cells induced a pronounced increase of NAD(P)H due to inhibition protons return into mitochondrial matrix (Pinelis et al., 2009; Surin et al., 2009). This effect of Oligo was considered as an indicator of normal OxPhos coupling. In the present experiments, we found that application of Oligo to embryonic neurons had no effect on NAD(P)H autofluorescence, suggesting OxPhos uncoupling (Figure 3B, blue trace; *P* > 0.05; *n* = 15). Another explanation of Oligo's failure to change NAD(P)H in these cells might be the low contribution of NAD(P)H to the recorded autofluorescence signal. To test this hypothesis, we used rotenone (Rot) to block complex I of the respiratory chain (blue bar in Figures 3A,B). Application of Rot (2 μM) induced a rapid increase in NAD(P)H autofluorescence (violet trace in Figure 3B) as well as rapid drop ΔΨm (red trace in Figures 3A,B), very similar to the effects of complex IV blockade by cyanide (Figure 2A; see also Appendix Figure A3). Thus, we concluded that mitochondrial respiratory chain in embryonic hippocampal neurons did not consume NADH for ATP synthesis. Together, the results shown in Figures 2 and 3 strongly suggest that the mitochondria of cultured embryonic hippocampal neurons used their respiratory chain to generate ΔΨm, but not to synthesize ATP, as their F1Fo-ATPase was functioning in a reverse mode (i.e., acted as an ATPase that hydrolyzed ATP).

One cannot exclude that embryonic cells may need a longer time to develop their metabolic phenotype as compared to postnatal ones, considering that they are approximately 6 days younger at the time when they are plated. To test this hypothesis, we measured the ratio between (1) the AT1.03 signal at the very beginning of the experiment and (2) the minimal ratiometric AT1.03 signal upon suppression of both glycolytic and mitochondrial ATP synthesis, and plotted this ratio against the days in culture (Figure 4A). We did not find any significant changes in this parameter during maturation of cultured neurons. The average values for young (5–8 DIV) and mature (13–14 DIV) cultures were 1.62 ± 0.11 (*n* = 39) and 1.66 ± 0.10 (*n* = 13), and showed no significant difference between them (*P* > 0.5, Figures 4A,B). In a separate series of experiments, we measured the ratiometric signals of the cytosolic AT1.03 at 8, 11, and 14 DIV (Figure 4C). In this case, we also failed to detect significant differences. Additionally, we compared the lag period and the rate of the [ATP]c drop following glucose deprivation. These kinetic parameters of the changes in [ATP]c in the mature cultures (filled squares in Figure 4D) were well within the range of values observed in young cultures (circles in Figure 4D). Only a minor fraction of neurons (17%, or 13/77 cells) in young cultures (5–8 DIV) showed a measurable increase in [ATP]c in response to Pyr addition to glucose-free buffer (Figure 4E). Cell culture maturation did not increase the proportion of neurons that were able to elevate [ATP]c in response to Pyr. In older cultures (13–16 DIV) the proportion of such neurons (Figure 4E) was even slightly lower (14%, or 5/37 cells).

**Figure A2:**
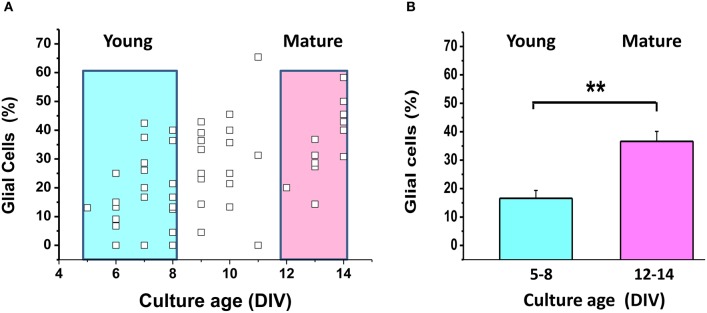
**The proportion of glial cells increases during maturation of mixed neuro-glial cultures. (A)** The small empty squares depict the proportion of glial cells (as % of all cells) in each of 57 fields of observation. Color boxes outline young (5–8 DIV) and mature (13–14 DIV) cultures. **(B)** Mean proportion of glial cells in young and mature cultures highlighted by color boxes in **(A)**. Identification of neurons and glial cells was performed as described in Abramov et al. (2003). Bright-field images and fluorescence images of Ca^2+^-sensitive indicators allowed the identification of neurons, which have a clearly distinct appearance as compared to the flatter glial cells and also lie in a different focal plane, above the glial layer. A total of 830 neurons and glial cells were analyzed in 50 experiments. ^**^p < 0.01.

It is well-known that glial cells play an important role in supporting neuronal structure and function both *in vitro* and *in vivo* [see for review Ullian et al. (2004)]. We found no significant difference between the signals of ATP sensor in young and mature neuronal cultures (Figure 4), although the proportion of glial cells relative to neurons increased from 16.6 ± 2.7% (5–8 DIV) to 36.6 ± 2.7% (13–14 DIV), as shown in Appendix Figure A2 (*P* < 0.01).

To test whether any peculiarities of our cultivation procedures may have accounted for these unexpected findings, we compared embryonic cultures to the postnatal ones (P1–P2) under the same conditions: transfected both cultures with AT1.03, loaded with TMRM and treated with Gluc-free buffer, 2-DG, Pyr and Oligo (Figure 5). We found that application of Gluc-free buffer containing 2-DG induced a much smaller [ATP]c decrease in postnatal neurons (by 30.1 ± 6.5%; *n* = 7) as compared to the near-complete depletion of [ATP]c (by 97.5%; see above) observed in embryonic ones (green traces in Figure 5C *vs.* 5A). This difference was significant (*P* < 0.05), further indicating that ATP synthesis in postnatal cultured neurons relied less on glycolysis and more on OxPhos than in embryonic neurons.

Furthermore, blockade of glycolysis resulted in a stronger ΔΨm decrease (i.e., larger rise in TMRM signal) in postnatal neurons compared to the embryonic ones (red traces in Figure 5C *vs.* 5A). On average, Gluc deprivation induced an increase in TMRM signal by 16.9 ± 6.0% in postnatal neurons (*n* = 18) which is significantly higher (*P* < 0.001) than in the embryonic ones (3.8%, see above). These data indicate that, when glycolytic ATP synthesis was blocked, mitochondria in postnatal neurons assumed the primary role in ATP production (thus causing stronger mitochondrial depolarization), which helped maintain [ATP]c on a higher level; in contrast, in the embryonic neurons the blockade of glycolysis quickly depleted [ATP]c due to reduced ability of their mitochondria to synthesize ATP by OxPhos.

Pyr addition to Gluc-deprived postnatal neurons also had a very different effect compared to embryonic cultures: it produced a marked increase in [ATP]c (Figure 5C, green trace) and a clearly measurable hyperpolarization of mitochondria (red trace). These effects of Pyr were significantly larger (*P* < 0.001) in postnatal neurons than in embryonic ones, as summarized in Figures 5E,G. Blockade of F1Fo-ATPase with Oligo addition to the Gluc-free buffer in the presence of extracellular Pyr induced a rapid and profound depletion of [ATP]c in postnatal neurons (Figure 5C), in sharp contrast to embryonic neurons (Figure 5A; statistics in Figure 5F). Effect of Oligo on ΔΨm in postnatal neurons was moderate but had an opposite direction (hyperpolarizing; Figures 5C,D, red traces) compared to embryonic neurons (depolarizing; Figures 5A,B, red traces). The difference in these effects of Oligo was highly significant (*P* < 0.001; Figure 5H).

In a subset of experiments, Oligo was applied prior to Gluc deprivation and glycolysis blockade and showed clearly distinct patterns of action on embryonic *vs.* postnatal cultures (Figure 5B *vs.* 5D). Thus, in embryonic neurons F1Fo-ATPases blockade produced mitochondrial depolarization but not [ATP]c depletion (Figure 5B), whereas in postnatal neurons it had little effect on ΔΨm but caused a profound decrease in [ATP]c (Figure 5D). On average, in embryonic neurons Oligo significantly decreased ΔΨm by 12.2 ± 2.4% (*P* < 0.001; *n* = 16) and increased [ATP]c insignificantly by 5.5 ± 2.8 (*P* > 0.05; *n* = 16). In contrast, in postnatal neurons Oligo induced a small increase in ΔΨm by 2.0 ± 1.3% (*P* > 0.2; *n* = 4) and a large decrease in [ATP]c by 66 ± 11% (*P* < 0.01; *n* = 8). Effects of Oligo were significantly different (*P* < 0.001) between embryonic and postnatal neurons, both in terms of ΔΨm and [ATP]c. Together, our findings demonstrate that the F1Fo-ATPase acted in two opposing modes in these two types of hippocampal cultures, mediating ATP synthesis in the postnatal neurons and ATP hydrolysis in most embryonic ones.

**Figure 6:**
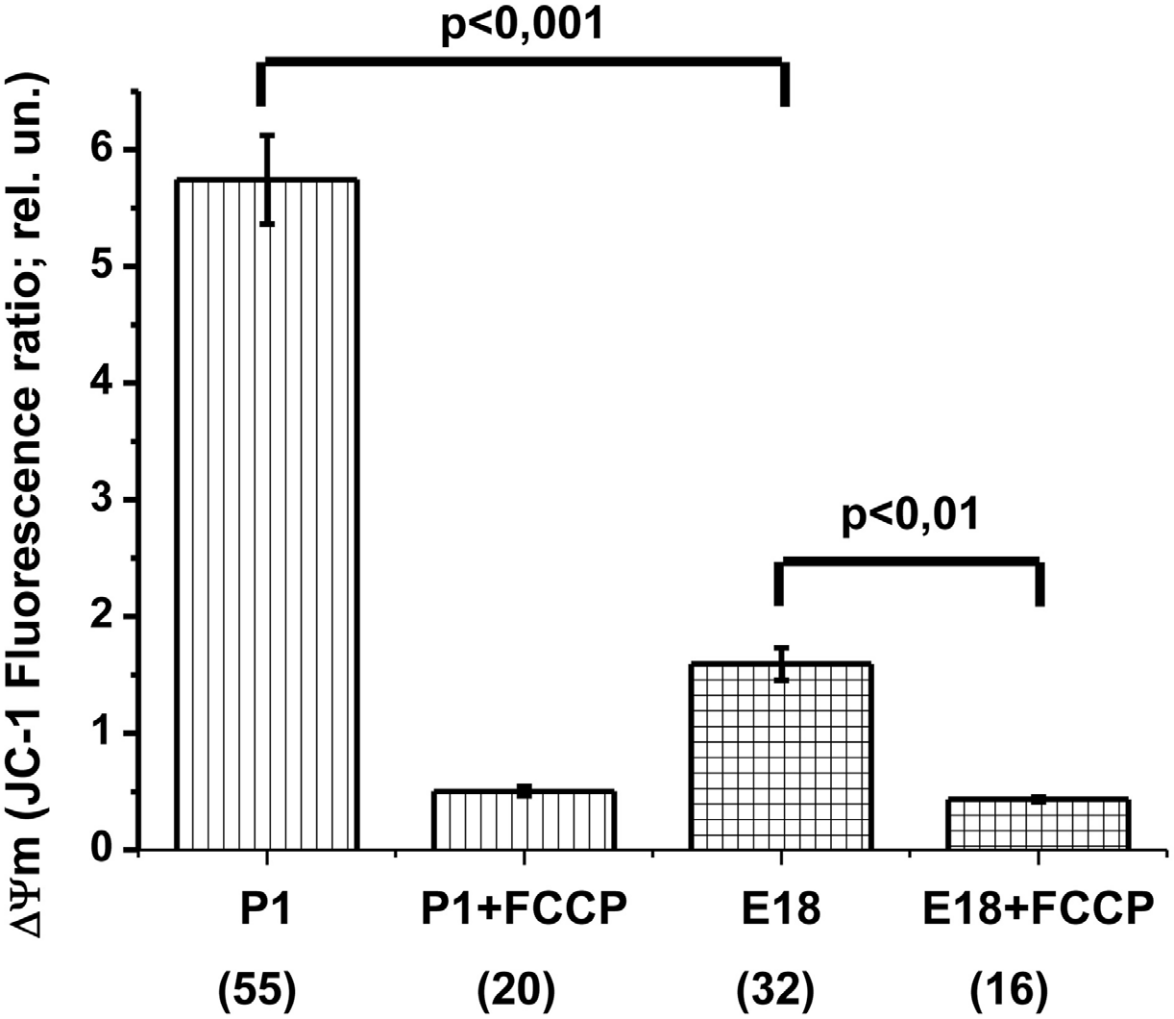
**Hippocampal neurons isolated from the embryos have a lower mitochondrial potential (ΔΨm) compared to neurons from newborn rats.** The values of ΔΨm as measured by the ratiometric probe JC-1, calculated as the ratio of fluorescence intensities at 640 ± 30 nm and 525 ± 20 nm (640/525), with excitation at 485 nm. Labels P1, P1 + FCCP, E18, and E18 + FCCP indicate postnatal (P1) and embryonic (E18) cultures before and after the protonophore FCCP (1 μM) addition. The number of cells utilized to prepare the histogram is shown in brackets.

In theory, several major reasons may account for the absence of OxPhos: (1) insufficient mitochondrial potential, which may lead to ATPsynthase reversal, (2) inhibition of the mitochondrial ATPsynthase and (3) blockade of ATP/ADP translocation. Oligomycin-induced [ATP]c increase revealed ATP translocation (Figures 3A,B and 5B), at least in one direction (i.e., into the matrix). The ATP synthase was also able to perform its work, at least in the reverse mode of ATP hydrolysis. Therefore, we focused on the mitochondrial membrane potential and compared ΔΨ_m_ of E17–E18 to that of P1–P2 hippocampal cultured neurons. To this end, we used the ratiometric potential-sensitive probe JC-1. Unfortunately, the interpretation of the JC-1 signal as such is ambiguous, especially in the case of kinetic measurements (Duchen et al., 2003). Therefore, we compared the JC-1 ratiometric signals in the soma of resting cells in embryonic *vs.* postnatal cultures. Both culture types were loaded with JC-1 under identical steady state conditions. Figure 6 shows that, in the culture of embryonic neurons, the ratiometric signal was only ~30% of that in cultured postnatal neurons. To verify the sensitivity of the JC-1 signals to ΔΨm, we added the protonophore FCCP (1 μM) at the end of the experiment. FCCP caused a collapse of ΔΨm, leading to an 11-fold decrease in JC-1 ratiometric signal in neurons from postnatal animals but only a 4-fold reduction in the neurons from embryos (Figure 6). Similar changes in JC-1 signal induced by FCCP have been reported for cortical neurons in culture (White and Reynolds, 1996). Together, these JC-1 experiments indicate that ΔΨ_m_ was much lower in embryonic neurons as compared to the postnatal ones.

## Discussion

The commonly accepted belief is that neuronal ATP is produced primarily by mitochondrial OxPhos and that glycolytic ATP synthesis plays a minor role in neurons (see for recent reviews, Cheng et al., 2010; Chinopoulos and Adam-Vizi, 2010; Shetty et al., 2012 and references therein). It was, therefore, a most surprising and unexpected finding of this work that ~84% (96/114) cultured embryonic hippocampal neurons, in contrast to their postnatal counterparts and in disagreement with the conventional view, failed to use their mitochondria to produce and maintain ATP.

This finding is supported by several lines of evidence we obtained from embryonic E17–E18 hippocampal neurons: (1) inhibition of glycolysis caused a near-complete depletion of [ATP]c, and the subsequent reversal of ATP synthase did not decrease [ATP]c any further (Figure 2A); (2) addition of mitochondrial substrates Pyr or Lac did not rescue OxPhos production of ATP to a degree that would measurably increase [ATP]c (Figures 2A,C, 3A,B, 5A,E); (3) inhibition of F1Fo-ATPase with Oligo in resting embryonic neurons did not affect [ATP]c and produced a depolarization, rather than hyperpolarization, of ΔΨm (Figure 5B); (4) during recovery from the[ATP]c depletion caused by Gluc deprivation, application of Oligo accelerated [ATP]c recovery, presumably because it blocked hydrolysis of ATP by the mitochondrial ATP synthase operating in a reverse, i.e., ATP consuming, mode. These results are summarized schematically in Table 1.

**Table 1:** **Comparison of the major differences of embryonic and postnatal hippocampal cultured neurons**.

**Pharmacological manipulation**	**[ATP]c**		**ΔΨ**_**m**_	
	**Embryonic**	**Postnatal**	**Embryonic**	**Postnatal**
Gluc deprivation	↓↓	↓	~	↓
Pyr (-Gluc)	~	↑↑	↑	↑↑
Oligo (-Gluc)	~	↓↓	↓	↑

Comparison between embryonic and postnatal cultured hippocampal neurons in terms of their responses to glycolysis blockade, supplementation of OxPhos ATP synthesis with Pyr, and F1Fo-ATPase blockade with Oligo. Two arrows indicate a strong change, one arrow indicates a moderate or weak change, the ~symbol indicates no change in a given parameter.

The unorthodox patterns of ATP synthesis observed in embryonic hippocampal neurons were not due to any peculiarities of our cultivation procedures, because the postnatal hippocampal neurons (which were cultivated in a very similar way; see section “Materials and Methods”) did not lose their expected ability to synthesize ATP by mitochondrial OxPhos (Figure 5). Indeed, inhibition of glycolysis in these P1–P2 cultured neurons (Figure 5C) decreased [ATP]c by only 30%, presumably due to the compensatory enhancement of ATP production by ATP synthase. Furthermore, addition of Pyr to Gluc-free buffer efficiently raised [ATP]c presumably due to a considerable mitochondrial hyperpolarization (Figures 5C,E,G). These kinds of responses to Pyr or Oligo were observed in all tested P1–P2 cultured neurons (6 experiments, 18 cells). Similar changes in ΔΨm and [ATP]c have been previously observed in cerebellar granular cells cultivated from P7 to P8 rat pups, as reported by our group earlier (Khodorov et al., 2012). Blockade of ATP production by Oligo in postnatal neurons induced, as a rule, a decrease in [ATP]c. However, the kinetics of this decrease varied in a wide range. Thus, in some cells [ATP]c declined soon after the beginning of Oligo challenge (see Figure 5D), while in other neurons a decrease of [ATP]c appeared only after a long-term Oligo application (20–30 min, not shown). We believe that these variations are mainly determined by the difference in the rate of ATP consumption and/or glycolysis rate during Oligo application between individual postnatal cells.

It is plausible that keeping the cells longer in culture may influence their metabolic parameters. However, the comparison between young and mature cultures of embryonic neurons revealed no change in [ATP]c dynamics (Figure 4). We investigated age-dependence of the ratio between (1) the AT1.03 signal at the very beginning of the experiment and (2) the minimal signal observed upon suppression of both glycolytic and mitochondrial ATP synthesis (Figures 4A,B). We did not find any significant change in this ratio between young (5–8 DIV) and mature (13–14 DIV) cultures. In a separate series of experiments, the ratiometric signals of AT1.03 at 8, 11 and 14 DIV were measured (Figure 4C). Here, we also failed to detect any significant differences between mature and young cultures. Additionally, we compared the lag period and the rate of [ATP]c fall after removing glucose from the buffer in the hippocampal neurons cultured from embryos. These kinetic parameters of [ATP]c changes in the mature cultures were well in the range of typical changes observed in young cultures (Figure 4D).

One of the major reasons for preparing neurons from embryonic tissue in our lab, as well as in many other groups, is to minimize the relative amount of glial cells in the cultures. We did not use cytostatics such as AraC to further suppress glia proliferation, because Lipofectamine manufacturer's guidelines do not recommend adding cytostatics to the cell culture medium as these toxic agents can reduce the efficiency of transfection and, in our case, of AT1.03 expression. The cytostatic-free conditions are comfortable for astroglia proliferation. *In vitro* studies revealed that astrocytes exert a powerful control over the number of CNS synapses, thus implicating astrocytes as participants in activity-dependent structural and functional synaptic changes (see for review Ullian et al., 2004). Astrocytes may provide neurons with lactate, supporting their needs for TCA substrates (Ames, 2000; Lust et al., 2003). We observed a 2-fold increase in the proportion of astrocytes relative to neurons in mature embryonic cultures compared to the young ones (Appendix Figure A2), but failed to detect a comparable increase in the proportion of neurons showing signs of OxPhos (Figure 4E). Taken together, our analysis suggests that keeping neurons longer in culture does not influence significantly those metabolic parameters that we have measured.

A question arises whether the data presented here are consistent with previously published results obtained by measuring [ATP] with biochemical or bioluminescent methods. To address this issue, we calculated the resting [ATP]c level in our cultured embryonic neurons. We based our calculations on the assumption that glycolysis blockade decreased [ATP]c to or beyond the minimal level detectable with AT1.03 (i.e., ~0.3 mM; Imamura et al., 2009). The dissociation constant of the complex AT1.03 with ATP and dynamic range of the ATP-sensor in the buffer, which mimics the cytosol salt composition, are 3.3 mM and ~2.5, respectively (Imamura et al., 2009). If both these parameters are valid for the cytosol, then the 1.6- to 1.7-fold decrease in AT1.03 signal following a complete blockade of glycolysis and mitochondrial ATP synthesis (Figures 4A,B) allows us to estimate the [ATP]c as ~2 mM. This value agrees well with [ATP] values obtained by biochemical methods in synaptosomes (~3 mM, Erecińska and Silver, 1989) and in the brain preparations (~4 mM, Lust et al., 2003). Similar [ATP] values (~1 mM) were calculated in cultured rat glucose-responsive neurons from hypothalamic nuclei, expressing luciferase (Ainscow et al., 2002; Mironov, 2007).

What are the cellular mechanism(s) underlying the observed inability of embryonic neurons to produce ATP by mitochondrial OxPhos? One obvious reason for this inability could be the malfunctioning of F1Fo-ATPase. The activity of this ATP synthase is known to be regulated by polypeptides such as the inhibitory factor IF1 (Pullman and Monroy, 1963; Klein et al., 1981) or the calcium-binding ATPase inhibitor protein CaBI (Yamada and Huzel, 1989). A plausible function for IF1 is to prevent conversion of ATP synthase into ATPase at low ΔΨm, thus preventing premature depletion of [ATP]c under conditions requiring active ATP consumption (Campanella et al., 2008, 2009). A somewhat similar role for CaBI may be to stimulate, in a Ca^2+^-dependent manner, ATP synthesis and inhibit ATP hydrolysis by F1Fo-ATPase (Yamada and Huzel, 1989). While we were not able to find any published evidence for developmental regulation of either IF1 or CaBI expression, our present data seem to indicate that in its reverse (ATP-hydrolysing) mode, the F1Fo-ATPase was functional in our embryonic hippocampal neurons (see Table 1). It seems plausible to suggest that F1Fo-ATPase may have been capable of ATP synthesis too, but was not functioning in this particular mode due to other reasons. It has been suggested, however, that transition from ATP-synthesizing mode to the ATP-hydrolysing one is not a mere reversal of the direction in which the Fo and central stalk rotary motor rotate against the F1 stator, but may involve distinct conformational changes (Vinogradov, 1999; Gao et al., 2005). Thus, we cannot rule out a possibility that F1Fo-ATPase was, indeed, dysfunctional specifically in its ATP-synthesizing mode. Further experiments would be required to test this hypothesis.

Another, possibly more likely, explanation for the observed lack of mitochondrial ATP synthesis in embryonic neurons is that their ΔΨm was too low to fuel OxPhos. In order to enable ATP synthesis by F1Fo-ATPase, the ΔΨm level must be above a certain level (Nicholls and Budd, 2000). Cortassa et al. (2003) have estimated that ATP synthesis is fully inhibited when ΔΨm becomes smaller than −100 mV (assuming that the pH difference across inner mitochondrial membrane is 0.4). Fluorescence imaging of ΔΨm-sensitive dyes (such as TMRM) in live cells has been combined with mathematical modeling to calibrate the measured fluorescence changes in terms of membrane potential changes in mV (Ward et al., 2000, 2007; Duchen et al., 2003; Nicholls, 2006). However, to the best of our knowledge, even for the most accurate calculations one needs to use in the equation an unknown, but commonly accepted initial value of ΔΨm. This value is usually assumed to be −150 to −180 mV, which is based on the measurements performed on isolated mitochondria because it cannot be directly measured in living cells (Ward et al., 2000, 2007; Duchen et al., 2003; Nicholls, 2006). We propose that, in our postnatal cultured neurons mitochondria were, indeed, highly charged (i.e., ΔΨm greater than −150 mV), whereas mitochondria of our embryonic neurons had a ΔΨm below the level required for OxPhos synthesis of ATP (i.e., near or even below −100 mV). The comparison of fluorescence signals of the potential-sensitive dye JC-1 in neuronal mitochondria of E17–E18 and P1–P2 rat pups yielded results that support this hypothesis (Figure 6). Such a low ΔΨm may be a result of lower efficiency of Pyr transport to mitochondrial matrix in embryonic neurons compared to the postnatal ones.

It is also possible that in embryonic cells the mode of operation of mitochondrial ATP syntheses is reversed due to a considerable increase in their proton conductance. Indeed, it is well-known that proton transport into mitochondrial matrix by free fatty acids plays a significant role in decreasing the ΔΨm, and that this process can be greatly accelerated by the uncoupling proteins shuttling fatty acid anions from the matrix to the cytoplasmic side of the inner mitochondrial membrane (see reviews by Ricquier and Bouillaud, 2000; Kim-Han and Dugan, 2005). Interestingly, the expression level of the brain-specific uncoupling protein subtype, UCP4, is high in mouse embryonic brain and gradually decreases during development (Smorodchenko et al., 2009).

The hypothesis of a considerably increased mitochondrial proton conductance in cultured neurons from embryonic rat hippocampus is based on the following data.

**Figure A3:**
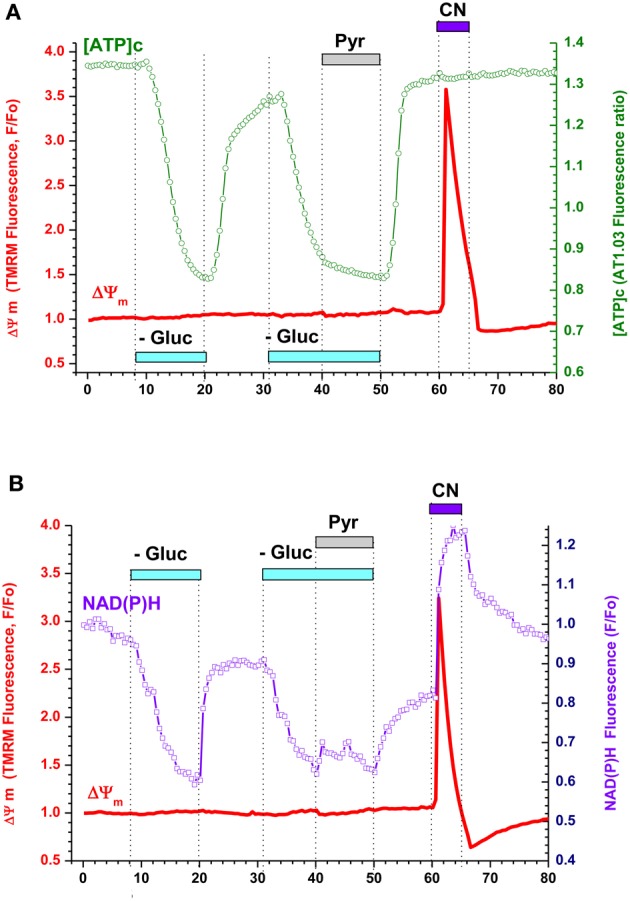
**Respiratory chain inhibition in neurons from embryonic rat hippocampus produced strong mitochondrial depolarization despite sufficiently high [ATP]c and NAD(P)H levels suggesting an increase in mitochondrial proton conductance. (A)** Simultaneous measurement of [ATP]c (green trace, right axis) and ΔΨ m (red trace, left axis) in neurons expressing AT1.03 and loaded with TMRM. Gluc-free buffer application is indicated with a light-blue bar, pyruvate (Pyr, 10 mM) with a gray bar, and cyanide (CN, 3 mM) with a dark blue bar. **(B)** Simultaneous measurement of averaged NAD(P)H autofluorescence (blue trace, right axis) and ΔΨ m (red trace, left axis) in two non-transfected neurons located in the same field of view as those shown in **(A)**. Pharmacological manipulations—same as in **(A)**.

1. As mentioned above (see section “Results”), application of Oligo to postnatal neurons induced a pronounced increase in mitochondrial NAD(P)H indicating the normal OxPhos coupling in these neurons(Pinelis et al., 2009; Surin et al., 2009). In contrast, in embryonic neurons Oligo failed to change NAD(P)H, suggesting the OxPhos uncoupling. Most likely, in cultured embryonic neurons in contrast to postnatal ones the rate of NAD(P)H consumption exceeds the rate of its production due to a considerable increase in mitochondrial proton conductance, therefore, Pyr addition failed to increase NAD(P)H during glucose deprivation (Figure 3B).
2. It is known that blockade of respiration in cultured postnatal cells by CN induces only a small mitochondrial depolarization due to reversal of mitochondrial ATP synthase (see, for example, Campanella et al., 2008). This occurs, however, only under two obligatory conditions: (1) proton conductance of the inner mitochondrial membrane should be small and (2) the [ATP]c should be sufficiently high to supply mitochondrial ATPase with ATP. We found that in embryonic neurons inhibition of respiration by NaCN (3 mM) produced, as a rule, a nearly maximal mitochondrial depolarization even at basal [ATP]c and sufficiently high NADH (Appendix Figure A3). This result strongly suggests that proton conductance in embryonic mitochondria exceeds that in mitochondria of postnatal cells.

The fact that in a Gluc-free buffer embryonic neurons were able to maintain their ΔΨm at a nearly constant level highlights several important issues. First, when Pyr is scarce, mitochondria apparently has an alternative source for substrate to support TCA and, therefore, respiratory chain. The most likely substitute for Pyr is glutamate (Glu) (Frigerio et al., 2008; Gellerich et al., 2010), which is abundant in cytosol where is reaches concentrations up to 10 mM (Nicholls, 2009). Glu is easily transported into mitochondria (Frigerio et al., 2008; Palmieri and Pierri, 2010), where it is rapidly oxidized to α-ketoglutaric acid, one of TCA substrates and products.

The second issue is that lowered, ΔΨm may have a protective function at the point of transition from placental existence to the life as an independent organism. At birth, animals experience a strong oxidative stress induced by the first intake of atmospheric oxygen. It is well-known that mitochondria are a major source of reactive oxygen species (ROS), and that mitochondrial ROS production is dramatically reduced upon lowering of ΔΨm (see reviews by Skulachev, 1996; Adam-Vizi and Chinopoulos, 2006; Chinopoulos and Adam-Vizi, 2006; Nicholls et al., 2007; Starkov, 2008). Considering that the brain consumes a disproportionally large amount of oxygen (which makes central neurons particularly vulnerable to the neonatal oxidative stress), one may suggest that reducing ΔΨm may be viewed as an efficient mechanism for protecting the fetus' neurons from the ROS-induced cell death. Along the same line of thinking, the fact that the OxPhos ATP production was predominant in hippocampal neurons cultivated from postnatal rat pups (Figure 4) suggests that newborn rats may have passed a crucial adaptation stage, which may have reduced their vulnerability to ROS. This notion is consistent with postnatal neurons having a ΔΨm sufficiently high for OxPhos synthesis of ATP. At birth, animals experience a strong increase in oxygen consumption induced by the first intake of atmospheric oxygen (Medina and Tabernero, 2005).

Finally, in a recent review by Thompson (2011) it was point out that “There are many eukaryotic species whose mitochondria lack the ability to carry out oxidative phosphorylation, but all require mitochondria and depend on mitochondrial anabolic biosynthesis. Once a cell is flooded with glucose and there are excess precursors, mitochondria can be reprogrammed into synthetic organelles.” One cannot exclude that, in the primary neuro-glial cultures from fetal brain, anabolic biosynthesis is a more important mitochondrial business than ATP production, at least in cultures prepared from the embryos on day 17–18 of their development.

## Materials and methods

### Preparation of rat hippocampal neurons

All experimental procedures were approved by the Animal Care and Use Committee, University of Helsinki and Lomonosov Moscow State University. Cultured neurons were prepared from embryonic day 17–18 (E17–E18) and newborn day 1–2 (P1–P2) rat hippocampi, as described earlier (Gorbacheva et al., 2008; Kolikova et al., 2011). Briefly, hippocampi were dissociated with papain solution (10 U/ml). The cells were plated at a density of (60–80) × 10^4^ cells/cm^2^ on glassbottomed Petri dishes (MatTek) pre-coated with poly-L-lysine and laminin (1–2 μg/cm^2^). Cultures were maintained in the 5% CO2/95% air atmosphere at 37°C in Neurobasal medium (Invitrogen; pH = 7.4) supplemented with B27 (Invitrogen), 0.5 mM L-glutamine, 100 U/ml penicillin, and 100 μg/ml streptomycin. Medium was changed by 1/3 of its volume (1.5–2 ml) twice per week.

### Cell culture transfection with plasmids

To monitor single-cell cytosolic [ATP] ([ATP]c) changes, the neurons were transfected with a plasmid encoding the ATP sensor AT1.03 (Imamura et al., 2009). For simultaneous monitoring of [ATP]c and pHc the plasmid encoding of red fluorescent protein pH-sensor mKate (Shcherbo et al., 2007) was added to the cells together with the AT1.03 plasmid. Neurons were transfected with DNA-constructs after 4–6 days *in vitro* using 2 μl Lipofectamine-2000 and 1 μg DNA per 250 μl of medium composed of 50 μl OptiMem and 200 μl of the cell transfection medium (both Invitrogen). Cell incubation medium was temporary removed leaving ~200 μl per well and 50 μl of DNA/Lipofectamin mixure was added into each well. The cells were stored in CO_2_-incubator at 37°C for 2–2.5 h, then medium was discarded, the cells were washed out by 2 × 1 ml of fresh Neurobasal medium and (temporary removed) conditioned medium was returned to the dishes. To determine the timing of the ATP sensor maturation, we relied on the fact that HeLa cells, expressing AT1.03 were used to measure [ATP] as early as on the first day after transfection (Imamura et al., 2009). A recent study described AT1.03 expression in hippocampal neurons from postnatal rats (Kovac et al., 2012). In that work, neuro-glial cultures were subjected to transfection at 11 DIV and used for fluorescence imaging after 1–2 days. Therefore, fluorescence analyses were carried out starting from DIV 5–7 (i.e., at least one day after transfection with AT1.03).

### Imaging of single cell [ATP]c, cytosolic pH and mitochondrial potential (ΔΨm)

Fluorescence microscopy measurements were performed employing the epifluorescent inverted microscope Olympus IX71 (Olympus Europe) with the 20×/0.70 NA or 40×/1.35 NA objectives at microscope stage temperature 32 ± 1°C. Fluorescence of AT1.03 was excited at 436 and 500 nm, and monitored at 535 nm using 510 nm beamsplitter. To measure TMRM fluorescence a 590 nm beamsplitter and excitation/emission filters centered at 575/640 nm were employed. Images were acquired by cooled CCD camera (Olympus DGH1, Japan or Photometrics, Cool Snap HQ) at 2 × 2 binning. Imaging data were collected and analyzed using the Cell-R software (Olympus, Japan) or MetaFluor 6.1 (Molecular Device, USA). Neuronal NAD(P)H autofuorescence was excitated at 360 ± 10 nm and monitored at 460 ± 20 nm. The values of ΔΨm as measured by the ratiometric probe JC-1, calculated as the ratio of fluorescence intensities at 640 ± 30 nm and 525 ± 20 nm (640/525), with excitation at 485 nm and a beamsplitter at 505 nm.

To acquire AT1.03 signals simultaneously with signals of potential sensitive dye TMRM, pH-indicator mKate or NAD(P)H autofuorescence we employed either rotary turret with dichroic cubes and emission filters mounted in them, or a tripleband beamsplitter with maximum reflections at 440, 490, and 570 nm and emission filter wheel in front of CCD camera. All filters and beamsplitters were from Omega (USA).

The cell fluorescence measurements were performed in a saline buffer containing (in mM): 130 NaCl, 5 KCl, 2 CaCl2, 1 MgSO4, 20 HEPES, 5 Glucose; pH 7.4. Where indicated, glucose was substituted by hexokinase inhibitor 2-deoxy-D-glucose (2-DG) (Pirolo and Allen, 1986). All agents were from Sigma-Aldrich (St. Louis, MO, USA). Membrane-permeable potential-sensitive dyes TMRM and JC-1 were purchased from Invitrogen (USA). TMRM presented in all buffers at concentration 40 nM. JC-1 (5 μg/ml) was loaded in cell incubation medium for 60 min at 37°C followed by rinse with saline buffer (2 × 1 ml) and fluorescence imaging.

### Data analysis

Images and graphs were constructed using the following software: AnalySIS (Olympus Europe), ImageJ (NIH), Origin (Microcal), Excel, and PowerPoint (Microsoft). Statistical analysis was performed using Origin and Excel.

### Conflict of interest statement

The authors declare that the research was conducted in the absence of any commercial or financial relationships that could be construed as a potential conflict of interest.
